# Perceived Stress Among Medical Doctors Working in Nepal: An Observational Study

**DOI:** 10.31729/jnma.v64i293.9291

**Published:** 2026-01-31

**Authors:** Lochan Karki, Anil Bikram Karki, Bikrant Dhakal, Aashutosh Chaudhary, Ashlesha Chaudhary, Suzit Bhusal

**Affiliations:** 1 Nepal Medical Association, Exhibition Road, Kathmandu, Nepal; 2 Kathmandu University School of Medical Sciences, Dhulikhel, Kavre, Nepal; 3 Everest Hospital Pvt Ltd, New Baneshwor, Kathmandu, Nepal; 4 Research and Development Unit, National Trauma Center, Mahankal, Kathmandu, Nepal

**Keywords:** *perceived stress*, *medical doctors*, *Nepal*, *occupational stress*, *mental health*, *PSS-10*

## Abstract

**Introduction:**

Physician stress has been a growing critical concern worldwide, including the Nepali doctors. However, in the post-COVID-19 era, the evidence on current stress levels remains limited.

**Methods:**

We conducted a cross-sectional survey between January and March 2024 among Nepal Medical Council-registered doctors working across Nepal. A web-based questionnaire including the 10-item Perceived Stress Scale (PSS-10) was used for data collection. Spearman’s coefficients were used to evaluate correlations, and t-tests and ANOVA were used to compare groups.

**Results:**

Among 302 medical doctors (median age 33 (IQR 28 to 41) years, 67.22% male), the mean PSS-10 score was 20.45±6.38. Overall, 43 (14.24%) had low stress, 205 (67.88%) had moderate stress, and 54 (17.88%) reported high stress. A higher PSS-10 score was associated with female gender, unmarried status, suicidal ideation/attempts, and family conflict. The PSS-10 scores of doctors in government hospitals were 21.80±6.43 and of those in teaching hospitals 18.71±6.49 (p=0.012). Compared to specialists (18.35±7.03), undergraduates and postgraduate physicians reported higher PSS-10 scores (21.61±6.15 and 20.62±6.05), and post hoc analyses confirmed significant differences (p<0.05). While social/family time was negatively correlated with PSS-10 scores (p<0.001), longer work hours were positively correlated with higher PSS-10 scores (p=0.008).

**Conclusions:**

Most participants reported moderate to high levels of stress, indicating a high prevalence of perceived stress among medical doctors in Nepal.

## INTRODUCTION

Perceived stress among healthcare professionals has become a critical concern in the past decade. Numerous studies in the last decade have documented a high prevalence of stress and burnout among physicians with global estimates ranging from 40%-80%.^1^"^3^ In Nepal, there are reports of almost 61.1-90% of doctors with at least moderate burnout levels and moderate to high levels of stress.^4,5^ Another study of 557 Nepali doctors reported prevalences of depression (25.41%), anxiety (30.89%), suicide risk (5.70%), and psychosomatic distress (20.50%), with moderate burnout risk.^6^

Although there have been reports of increasing stress among Nepali physicians, there is a significant gap in terms of the extent and factors contributing to the stress in Nepal, particularly in the post-COVID pandemic era. This study aims to address this gap by studying perceived stress levels among Nepali doctors and identifying key contributors in the current context.

## METHODS

A nationwide cross-sectional study was conducted between January and March 2024 in government, private, and teaching hospitals across Nepal. Our study population included paid working Nepal Medical Council-registered doctors, including those employed by hospitals, clinics, or private practices, as well as those who are self-employed in Nepal. Medical doctors were eligible if they possessed at least a Bachelor of Medicine and Bachelor of Surgery (MBBS) or a Bachelor of Dental Surgery (BDS) degree and were engaged in clinical or academic practice at the time of data collection. Potential respondents who did not meet these criteria or who did not complete essential sections of the survey were excluded.

Data collection took place over approximately three months. Ethical approval was obtained from the Nepal Health Research Council, and the study was conducted in strict adherence to relevant national guidelines governing research. Data collection was started only after obtaining ethical approval. Assuming that 85% of the subjects in the population have the outcome of interest, based on a previous study^4^ and assuming a response rate of 65% to account for potential non-response, the study would require a sample size of 302 for estimating the expected proportion with 5% absolute precision and 95% confidence.

A web-based data collection approach was undertaken to maximize reach, considering Nepal’s geographical diversity and the varying nature of medical practice. Data collection was performed through online distribution of the questionnaire using Google Forms, following the non-probability sampling method. The questionnaire was distributed to relevant social media groups targeting the inclusion of medical doctors across all states of Nepal. A large proportion of doctors in Nepal mainly work in government and private hospitals and also in academic settings in teaching hospitals, which can be both government and private.

We distributed our questions so that we involved doctors working in each setting across each province in Nepal. We also asked the individuals who received the questionnaire to forward it to their colleagues in their respective institutions in the country. Responses were regularly monitored to check for discrepancies. Since the questionnaire was distributed online, while beneficial for reaching diverse participants, it precluded the possibility of an accurate response rate calculation, as the total number of individuals who accessed the link could not be tracked.

The online questionnaire included a section of the participant information sheet explaining voluntary and anonymous participation. Informed consent was obtained through the online survey itself. The participants were not asked for any identifying information, maintaining confidentiality. Individuals could exit the survey at any time without providing a reason. The participants received no enumeration or financial incentives for their participation.

Stress levels were measured using the Perceived Stress Scale-10 (PSS-10), a validated tool for assessing perceived stress.^7^ This scale has been extensively used in medical research, and its reliability, validity, and psychometric properties have been studied extensively across different populations with acceptable results.^7-9^ Also, the scale has been used in previous studies involving Nepali healthcare professionals.^4,5^ Due to the questionnaire’s strong psychometric properties and diverse applicability across different populations, this scale has also been registered for use in the National Institutes of Health’s Toolbox for the Assessment of Neurological and Behavioral Function (i.e., NIH Toolbox).^10^ Because of these reasons, we decided to use this scale for our study.

This self-report questionnaire evaluates how much a respondent perceives their life circumstances as overwhelming, unpredictable, and/or beyond their control. The PSS records a respondent’s subjective assessment of whether current circumstances and life events exceed their adaptive capability.

The PSS asks respondents to rank their thoughts and feelings over the last month, suggesting that a reasonably recent period is evaluated.^7,9^

This scale consists of 10 questions where participants respond based on a Likert scale. Scores ranging from 0 to 13 are categorized as low stress, scores from 14 to 26 as moderate stress, and scores from 27 to 40 as high perceived stress. Confirmatory factor analysis of PSS-10, done by Taylor J.M., has revealed that the two-factor model best explains the relationship between the items.^8^ The two subscales include the Perceived Self-Efficacy Subscale, which includes the negatively worded items, i.e., Q4, Q5, Q7, and Q8, and the Perceived Helplessness Subscale, which includes the rest of the items. With questions on demographic characteristics, work-life balance, and health status, and the PSS-10 scale, we pre-tested on a small group of volunteer doctors (n=10) before starting the main data collection to ensure clarity of the questionnaire, which led to only minor refinement while scale items remained unchanged, and the responses from the pilot group were not included in the final analysis.

Survey responses were stored automatically in a secure online repository, and access to these data was limited to the principal investigator and authorized team members. The data was exported from Google Form. The data were then imported into Jamovi (The Jamovi Project (2024), version 2.6) and coded. Data was checked for any anomalies and missing values. The data was checked for normality using histograms, density plots, QQ plots, and the Shapiro-Wilk test. The responses from the PSS-10 scale were observed and checked for any anomalies. The scores for questions 4, 5, 7, and 8 were reversed as recommended. Then, the total PSS-10 score was obtained by adding up the scores from each item.

Descriptive analyses (including frequencies, percentages, means with standard deviations, and medians with interquartile ranges) were used to summarize participant characteristics. The choice of statistical tests was guided by assessments of normality. Independent samples t-tests and one-way analyses of variance (ANOVA) were performed to analyze differences in mean PSS-10 scores across groups. Tukey’s post hoc comparisons were conducted for the ANOVA results. Spearman’s correlation coefficients were used to explore the correlation between the PSS-10 score and continuous variables in our study (e.g., age, working hours, time spent with family).

All participants provided electronic informed consent before completion of the questionnaire. Study protocols followed ethical standards in accordance with national guidelines. Data was maintained and protected on secure servers and accessible only to the principal investigator and research team members directly involved in analysis.

## RESULTS

A total of 302 medical doctors participated in the study. Among them, 203 (67.22%) were male, 96 (31.79%) were female, and 3 (0.99%) preferred not to disclose their gender. The mean and median age of the participants was 35.54±9.07 years and 33.00 (IQR 28 to 41) years respectively. The dominant age group of our participants was below 30 years and between 30 and 40 years, with 107 (35.43%) and 113 (37.42%) respondents, respectively(Table 1).

**Table 1:** Sociodemographic details of the study population (n=302).

Details	n(%)
Gender	
Male	203(67.22)
Female	96(31.79)
Prefer not to disclose	3(0.99)
Age group	
Below 30	107(35.43)
30-40	113(37.42)
40-50	65(21.52)
50-60	10(3.31)
60-70	6(1.99)
70 and above	1(0.33)
Academic level	
Undergraduate (MBBS/BDS)	93(30.79)
Postgraduate (MD/MS/MDS)	146(48.34)
Specialty Training (DM/MCh/	63(20.86)
Fellowships)	
Work place	
Government Hospitals	118(39.07)
Teaching Hospitals	83(27.48)
Private Hospitals	76(25.17)
Other Healthcare Settings	25(8.28)

The median working hours per day were 9.00 (IQR 8.00 to 12.00) hours, while the median time spent with family and friends per day was 3.50 (IQR 2.00 to 5.88) hours. Participants reported different levels of work-life balance and the influence of work on personal relationships (Table 2).

**Table 2:** Descriptive statistics for work-life balance, relationship impact, and daily time allocation (n=302).

Factors	n(%)
Work-Life Balance	
Very Balanced	11(3.64)
Balanced	68(22.52)
Neither Balanced nor Imbalanced	109(36.09)
Imbalanced	73(24.17)
Very Imbalanced	41(13.58)
Work Impact on Relationships	
Never	83(27.48)
Sometimes	154(50.99)
Frequently	65(21.52)

**Reliability analysis**

The reliability analysis shows that the scale has a McDonald’s ro of 0.8675 and a Cronbach’s a of 0.8658. Item-level statistics showed that all items have acceptable item-rest correlations (ranging from 0.39 to 0.73) and that removing any one item does not significantly improve overall reliability (a remains in the range of 0.844-0.867).

**Level of Perceived Stress**

Our primary objective was to determine the stress level in our study population using the PSS-10 scale. Our population’s mean perceived stress scale-10 score was 20.45±6.38. Regarding stress levels, 43 (14.24%) of the participants reported low stress, 205 (67.88%) experienced moderate stress, and 54 (17.88%) reported high stress levels.

**Figure 1:**
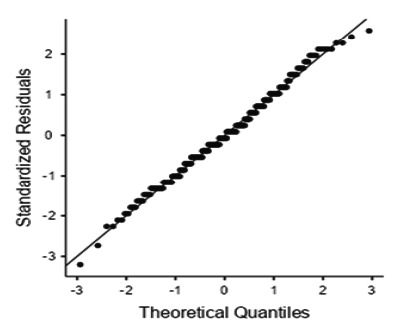
Quantile-quantile (Q-Q) plot of Perceived Stress Scale (PSS-10) total scores.

Moderate level of stress was present in 66 (70.97%) undergraduate, 98 (67.12%) postgraduate and 41 (65.08) speciality level training participants (Table 3).

**Table 3:** Level of Stress amongst different levels of completed training (n=302).

Level of training	Low Stress n(%)	Moderate Stress n(%)	High Stress n(%)
Undergraduate (MBBS/BDS)	9(9.68)	66(70.97)	18(19.35)
Postgraduate (MD/MS/MDS)	20(13.70)	98(67.12)	28(19.18)
Specialty Training (DM/MCh/Fellowships)	14(22.22)	41(65.08)	8(12.70)

Independent samples t-tests showed that PSS-10 score was 22.95±6.01 in female and 19.11±6.09 in male, p-value <0.001). PSS-10 score was 25.67±5.40 in individuals with suicidal ideation, 24.70±4.92 in individuals with family conflicts and 23.20±4.92 in unmarried (Table 4).

**Table 4:** Results of the Independent Samples T-Test between PSS-10 scores and other variables.

Analysis	Group	n	Mean±SD	p-value	Effect Size (Cohen’s d)	Shapiro-Wilk test (p-value)
Gender	Female	96	22.95±6.01	<0.001	0.63	0.421
	Male	203	19.11±6.09			0.345
Suicidal Ideation	Yes	39	25.67±5.40	<0.001	0.99	0.072
	No	263	19.68±6.16			0.430
Family Conflicts	Yes	33	24.70±4.92	0.007	0.58	0.062
	No	269	19.93±6.36			0.177
Marital Status	Unmarried	25	23.20±4.92	<0.001	0.55	0.450
	Married	229	19.58±6.41			0.061

**Table 5:** Results of ANOVA between PSS-10 scores and other variables.

Analysis	Group	Mean (SD)	F	p-value	Effect Size	Shapiro-Wilk(p-value)	Significant Post Hoc Differences
Academic Level	Undergraduate (MBBS/BDS)	21.61±6.15	5.14	0.006	ω^2^=0.027	0.585	vs. Specialty: p=0.005
	Postgraduate (MD/MS/MDS)	20.62±6.05				0.350	vs. Specialty: p=0.045
	Specialty Training (DM/MCh/Fellowships)	18.35±7.03				0.210	
Work setting	Government Hospitals	21.80±6.43	3.85	0.010	ω^2^=0.028	0.844	vs. Teaching: p=0.012
	Teaching Hospitals	18.71±6.49				0.108	
	Private Hospitals	20.93±5.69				0.432	
	Other Healthcare Settings	19.04±7.83				0.268	
Illness Status	No Illness	20.20±6.38	3.17	0.044	ω^2^=0.014	0.134	vs. Psychiatric: p=0.033
	Medical Condition	20.29±5.91				0.164	
	Psychiatric Condition	23.90±6.24				0.382	

A one-way ANOVA indicated differences in PSS-10 scores by academic discipline (p=0.006) (Table 5), with undergraduates reporting the mean stress 21.61±6.15.

Regarding health status, participants with psychiatric conditions reported mean stress 23.90±6.24.

Tukey post hoc tests indicated a significant difference between the no illness and psychiatric groups (p = 0.033)(Table 5).

**Correlation Analysis**

Age, time spent at work, and time spent with family or friends were not normally distributed (all Shapiro-Wilk p<0.001). A Spearman correlation analysis revealed a significant correlation between PSS-10 scores and factors including age, time spent with family and friends, and time spent at work. The PSS-10 showed a negative correlation with age (r=-0.2783, p<0.001).

Time spent with family and friends was inversely connected with total PSS-10 (r=-0.2905, p <.001), time spent at work had a positive correlation with total PSS-10 (r=0.1524, p=0.008).

## DISCUSSION

We found a significant stress-related concern among medical doctors, with 67.88% experiencing moderate stress and 17.88% reporting high-stress levels. Female doctors had significantly higher PSS-10 scores than males (22.95± 6.01 vs 19.11±6.09, p<0.001). Those with a history of suicide attempts/suicidal ideation exhibited higher PSS-10 scores than those without (25.67±5.40 vs 19.68±6.16, p<.001). Unmarried doctors reported greater PSS-10 scores than married ones (22.50±6.11 vs 19.10±6.22, p<.001), while PSS-10 scores were highest among undergraduates (21.61±6.15), followed by postgraduates (20.62±6.05) and specialty trainees (18.35±7.03).

In a study conducted among 380 healthcare professionals, including doctors, nurses, and other allied health workers during the pandemic, around 90% of the healthcare workers experienced moderate to high perceived stress in Nepal.^5^ Another study conducted among doctors and nurses during the pandemic reported that around 85% of participants had scores suggesting moderate to high perceived stress.^4^ Both of these studies used the same perceived stress scale(PSS-10).^4,5^ In our study, we had a similar finding where around 85.76% of the doctors experienced moderate to high perceived stress. This can indicate that stress is still a matter of great concern for healthcare professionals in Nepal, even beyond the pandemic.

A 2021 survey of Nepalese resident doctors during COVID-19 (using the DASS-21 stress scale) reported an 8.2% prevalence of stress above the clinical threshold.^11^ This figure appears low relative to other studies, likely due to strict criteria for “stress” in that instrument or sample specifics (many may have reported mild stress that didn’t meet the cutoff). A study conducted among 555 residents(those with ongoing postgraduate studies) in Jordan using the Perceived Stress Scale-10 found that 73% had moderate stress levels, and 18% had high stress levels.^12^ In our study, medical doctors from different walks of life also reported similar perceived stress levels, with 67.88% experiencing moderate stress and 17.88% reporting high stress levels. We also found that undergraduate doctors (medical officers and residents) in our study had the highest level of perceived stress. In contrast, those who had completed specialty training had the lowest levels of perceived stress. Medical officers and residents tend to have long working hours with lower pay. Also, since they are yet to complete their education, they have the burden of academics and strong competition. In the study by Maswadi et al., factors strongly associated with stress among residents were female gender, work environment dissatisfaction, and academic and family stressors.^12^

Female doctors had significantly higher stress levels (M=22.95, SD=6.01) than males (M=19.11, SD=6.09), t(297)=5.10, p<.001. This is consistent with previous literature where female doctors report higher perceived stress with worse psychological domains such as burnout and depression.^13-16^ In the context of Nepal, added domestic responsibility and social expectations could have led to this gender-based discrepancy.

Unmarried doctors reported greater stress than married ones (22.50±6.11 vs 19.10±6.22, p<.001). This is consistent with previous literature where unmarried status in physicians was associated with worse psychological outcomes, especially during the COVID-19 pandemic, and marriage has also been considered a protective factor against physician burnout.^17-19^ Lack of family support could be a contributing factor. Also in our study, time spent with family members negatively correlated with PSS-10 scores, which may support this assumption.

Working hours positively correlated with PSS-10 (r=0.122, p=.034). Working longer hours (defined as>8 hours per day/>40 hours per week) is associated with negative health outcomes such as depressive state, anxiety, truncated/disturbed sleep, and coronary heart disease.^20^ In our study, more than 50% of the participants worked more than 8 hours per day, and 223 (73.84%) of the participants didn’t consider having a proper work-life balance. Time spent with family members was negatively correlated with work-life balance.

In our study, 39 (12.91%) reported a history of suicide attempts/or suicidal ideations. Participants with a history of suicide attempts/suicidal ideations had significantly higher PSS-10 scores than those without (25.67±5.40 vs 19.68±6.16, p<.001). This is consistent with findings from previous studies where higher PSS scale scores were independently associated with a higher risk for suicidal ideation.^21^ Thus, symptoms of stress must warrant evaluation of suicidal ideation, and measures to reduce stress and stress management can be crucial to decrease the burden of suicide in this at-risk population.^22,23^

The need for focused solutions to support the well-being of medical professionals is highlighted by the high levels of stress among them. Stress levels are elevated by long working hours and limited time for social and family activities. To reduce the effects of workplace stressors, hospitals and other medical facilities should think about introducing stress management programs, flexible work hours, and mental health support.

There are certain limitations in our study. We were not able to determine the response rate of the survey because it was conducted online, and we couldn’t determine how many individuals received the questionnaire. Since the survey distribution was done through professional and institutional networks, doctors who work in rural, remote areas, and those practicing in smaller practice settings may be underrepresented in the study. Since this study was conducted through an online self-reported survey, there could have been misclassifications in the levels of stress, and there is a risk of response bias. Post hoc analysis was performed to explore associations beyond the primary outcome. These results may be subject to limited statistical power, and they should be interpreted with caution as exploratory.

## CONCLUSION

Perceived stress is a considerable concern among medical doctors in Nepal, with most doctors experiencing moderate to high stress. The findings align with known worldwide studies reported in which occupational and systemic factors have a major impact on stress levels. Younger, female, or unmarried physicians, those in training or teaching hospitals, and doctors with psychiatric conditions or suicidal ideation showed higher stress levels.

## Data Availability

The authors confirm that data supporting the findings are available within the article and its supplementary materials.
